# Three-Dimensional Printing in Cardiothoracic Surgery: Workflow and Clinical Applications

**DOI:** 10.7759/cureus.85530

**Published:** 2025-06-07

**Authors:** Charles G Jenkinson, Tristan L Wood, Jason Chuen

**Affiliations:** 1 Department of Cardiothoracic Surgery, Prince of Wales Hospital, Sydney, AUS; 2 Faculty of Medicine, University of Western Australia, Perth, AUS; 3 Faculty of Medicine and Dentistry, Charles Sturt University, Orange, AUS; 4 Department of Cardiothoracic Surgery, Sir Charles Gairdner Hospital, Perth, AUS; 5 Vascular Surgery, Austin Health, Heidelberg, AUS; 6 Vascular Surgery, Melbourne University, Melbourne, AUS

**Keywords:** 3d printing in surgery, 3d printing technique, patient consent, student education, three-dimensional (3d) printing

## Abstract

Rapid advances have been made in the field of three-dimensional (3D) printing in recent times. The hardware required to create high-fidelity, anatomically accurate 3D models of intrathoracic structures, including the great vessels and bony structures, is accessible and easy to use and utilizes consumables that are cost-effective. The quality of 3D printers, especially in consumer-level machines, has improved in recent times, while the price of the hardware has decreased. We have developed a toolchain to create 3D-printed models for use in patient education and consent, operative planning, and student/resident teaching. We outline our approach using a free and open-source toolchain, highlighting the utility of our models so that others may reproduce our techniques in their own clinical and teaching practice.

## Introduction

Three-dimensional (3D) printing is rapidly gaining traction in cardiothoracic surgery as a versatile tool for improving anatomical visualisation, surgical planning, and education. By translating patient-specific imaging data into tangible models, 3D printing enables surgeons to better understand complex pathologies, select optimal operative strategies, and communicate effectively with patients, residents, and students. Although augmented reality and virtual reality solutions have been developed, 3D-printed models provide excellent resolution and tactile feedback - practitioners, patients, or students can touch, feel, and physically move the structures in real space [1]. Developments in materials have allowed practitioners and residents to rehearse surgery or procedures on higher-fidelity models than previously available.

A variety of 3D printing technologies exist, including fused deposition modelling (FDM), stereolithography (SLA), digital light processing (DLP), selective laser sintering (SLS), photopolymer jetting, powder binder printing, and 3D laser bioprinting (LAB). The most common 3D printing modalities seen in clinical use are FDM and SLA. Both technologies build objects layer by layer. FDM prints with adhesive materials such as thermoplastic filaments, including polylactic acetate (PLA) and acrylonitrile butadiene styrene (ABS). It is cost-effective and well-suited for producing larger anatomical structures, such as the entire aortas or bony structures, intended for consent discussions, operative planning, or general teaching purposes [2-4]. By contrast, SLA uses a focused light source to cure photopolymer resins with high precision, producing highly detailed models. SLA’s ability to use biocompatible materials also makes it suitable for intraoperative applications, such as sterilised sternal models for plate pre-bending [5].

Previous studies have demonstrated that 3D-printed cardiovascular models improve preoperative planning by allowing better assessment of vessel geometry and planning of grafts and other surgical interventions [6-8]. Other work highlights the benefits of customised chest wall models for reconstructive planning after sternal wound breakdown or resection [5,9]. As access to human anatomical specimens declines, printed models of ribs, particularly the first ribs, serve an increasingly important role in anatomical education and simulation [4].

We outline our clinical workflow and experience with 3D printing in a cardiothoracic surgery unit, with applications spanning patient consent for aortic procedures, anatomical teaching, operative planning, and reconstruction. We have previously described our methodology for the segmentation of CT datasets for the purposes of computational fluid dynamics assessment of flow characteristics in aortic aneurysm [10]. The production of 3D printable models shares much in common with the initial stages of this previous work.

## Technical report

Image acquisition 

3D printing begins with a high-quality dataset on which a CAD (computer-aided design) model is based. Patients are sent for CT scans of suitable quality. As we described when outlining our workflow for computational fluid dynamics from CT datasets, good quality images are required [10]. Tomographic slices of 0.625 mm or less are ideal (although we prefer 0.5 mm slice thickness) and should minimise step off, bloom, or streak artefact [11].

For the imaging of the aorta and/or pulmonary vessels, appropriately phased contrast-enhanced images should be obtained, ECG-gated in diastole. For aortic imaging, acquisition should extend from at least the root of the neck to the diaphragm to ensure that the entire thoracic aorta is obtained. If only the aorta is to be printed, minimisation of contrast in the venous structures (especially the nearby brachiocephalic vein) improves the accuracy of segmentation and reduces the time taken in subsequent processing steps [10]. Imaging of bony structures can be achieved with non-contrast, cardiac-gated images with thin slices.

We obtain imaging from a variety of radiology practices, including from our own hospital radiology department, to an array of private external companies. In our experience, engagement with an interested radiologist with thoracic expertise is a useful step, and often improves the accuracy and technical quality of subsequent imaging. A variety of CT scanners are used to create these images, which are uploaded to our institution’s Picture Archiving and Communication System (PACS) server as industry-standard Digital Imaging and Communications in Medicine (DICOM) files.

Occasionally, we use imaging purely for teaching purposes. The Database Center for Life Sciences at the National Institute of Genetics, Yata, Japan, contains an atlas of human anatomy, including the ability to export files under a Creative Commons Attribution-Share Alike 2.1 license (CC BY-SA 2.1 CA; https://creativecommons.org/licenses/by-sa/2.1/ca/) [12]. We have used this to create real-sized 3D prints of the heart and first rib.

Image review and de-identification 

Horos (version 3.3.6, Horos Project, Annapolis, MD, USA, 2024), running on MacOS 15 Sequoia (version 15.4.1, Apple Inc., Cupertino, CA, USA, 2025) is used to review CT images stored in DICOM format for technical quality and allows the images to be stripped of any identifying metadata. This ensures patient privacy in the future. An alternative cross-platform solution is 3D Slicer (http://www.slicer.org) [13].

Segmentation 

**Figure 1:**
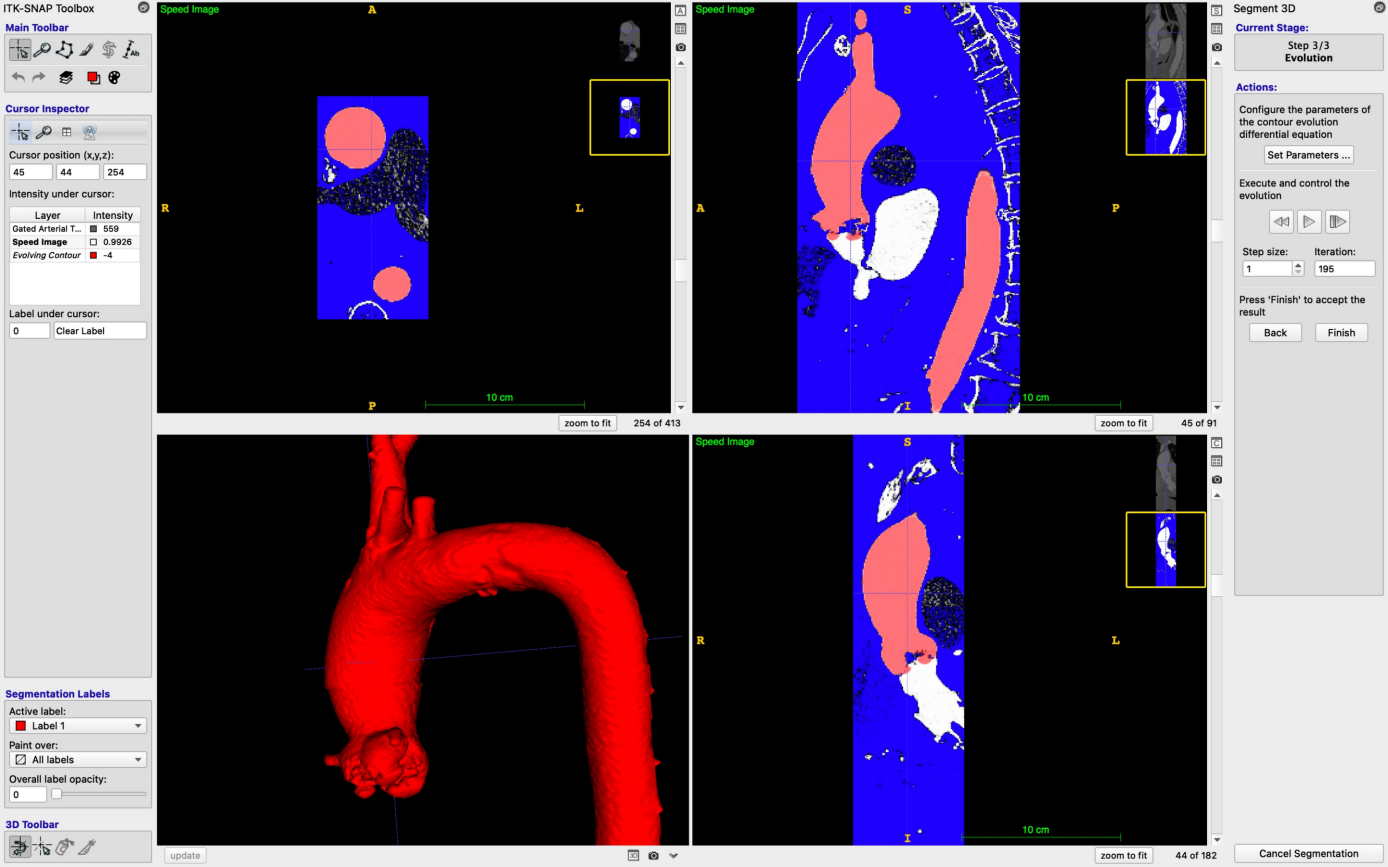
An aorta undergoing active contour segmentation in ITK-SNAP to generate a mesh for further processing and 3D printing. The figure is the authors' own.

For segmentation of bone or contrast within blood vessels, ITK-SNAP (version 4.2.2, University of Pennsylvania, Philadelphia, PA, USA, https://www.itksnap.org, 2024) is a cross-platform, free, and open-source software (FOSS) package commonly used in medical research. It supports various imaging formats, including Neuroimaging Informatics Technology Initiative (NIfTI) and DICOM, and offers features like linked 3D navigation, manual and automatic segmentation, and other advanced algorithms. ITK-SNAP is used to create a 3D mesh in .stl (stereolithography) format [14]. An STL file is a 3D model format that encodes surface geometry as a mesh of triangles, commonly used for slicing and printing in 3D fabrication. Semi-automatic segmentation is carried out in "active contour segmentation mode" with Hounsfield unit (HU) gating from approximately 300 to 900, as we have previously described [10]. This is illustrated in Figure 1.

A .stl file is exported from ITK-SNAP for further processing. 

Image cleaning and modification 

A copy of the .stl file exported from ITK-SNAP is then imported into Blender (version 4.4.3, Blender Foundation, Amsterdam, Netherlands, https://www.blender.org, 2025). By selecting the mesh(s) of interest, usually vascular structures or bone, the selection may then be inverted, and artefactual meshes are deleted, ensuring that only the structures of interest are captured. The mesh may also be trimmed, if necessary, for example, to remove excess lengths of vessels, including coronary arteries that do not add useful information to the model. This is then exported once more as a .stl file.

Blender can also create 3D stands for holding the structures in anatomical location or supporting the model in an anatomical position. This adds complexity to our workflow and is seldom needed in our experience.

Image smoothing 

The .stl file resulting from this process encodes a series of cubes (voxels), which match the size of the CT scan’s slice thickness and are often not “closed” or “bound”. MeshLab is a FOSS application that allows filtering of a 3D mesh to improve smoothness and ensure that a mesh is fully closed [10]. A screened Poisson surface redistribution is used to achieve this [15]. The resulting mesh is exported as a .stl file for final printing using default settings of reconstruction depth = 8, interpolation weight = 4, minimum number of samples = 1.5, scale factor = 1.1, solver divide = 8, iso divide = 8, and smoothing iterations = 5.

3D printing 

The final .stl file is then imported into our 3D printer’s software. For vascular and bony structures used either for consent or medical education, we print on a consumer-grade FDM 3D printer (Flashforge Adventurer 3 3D Printer, Zhejiang Flashforge 3D Technology Co., Ltd., https://flashforge-usa.com/products/adventurer-3, China). This printer uses Flashprint (version 5.8.7, Zhejiang Flashforge 3D Technology Co., Ltd., China) and allows the automatic creation of rafts and supports, as well as scaling. Rafts are a temporary flat bed of printing material, designed to improve adhesion of the model to the print bed. Supports temporarily hold up a structure (such as the aortic arch) to enable material deposition at an overhang. We have found the ability to enlarge an area of interest to be useful, especially when printing models of congenital heart disease, such as aberrant coronary arteries.

**Figure 2:**
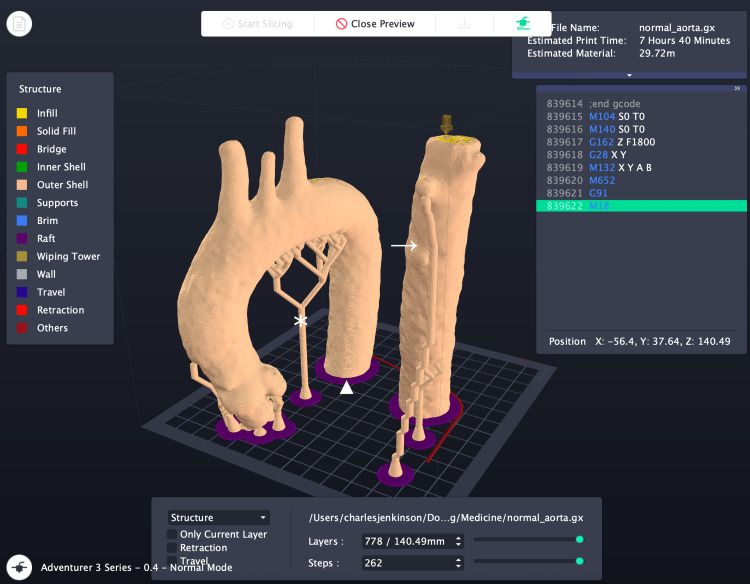
An anatomically normal aorta set up to print in FlashPrint. Note the presence of supports and rafts. The descending thoracic aorta has been divided to allow the print to proceed in the 15 x 15 x 15 cm print area and is subsequently joined to the other component with cyanoacrylate glue. ★ Indicates *temporary support structures* used to maintain overhanging geometry during printing. ▲ Denotes the *raft*, a foundational base layer printed beneath the model to improve bed adhesion and reduce warping. → Signifies the *descending aorta* printed separately, for attachment to the main model after removal of support structures and rafts. The figure is the authors' own.

The setup process is shown in Figure 2.

**Figure 3:**
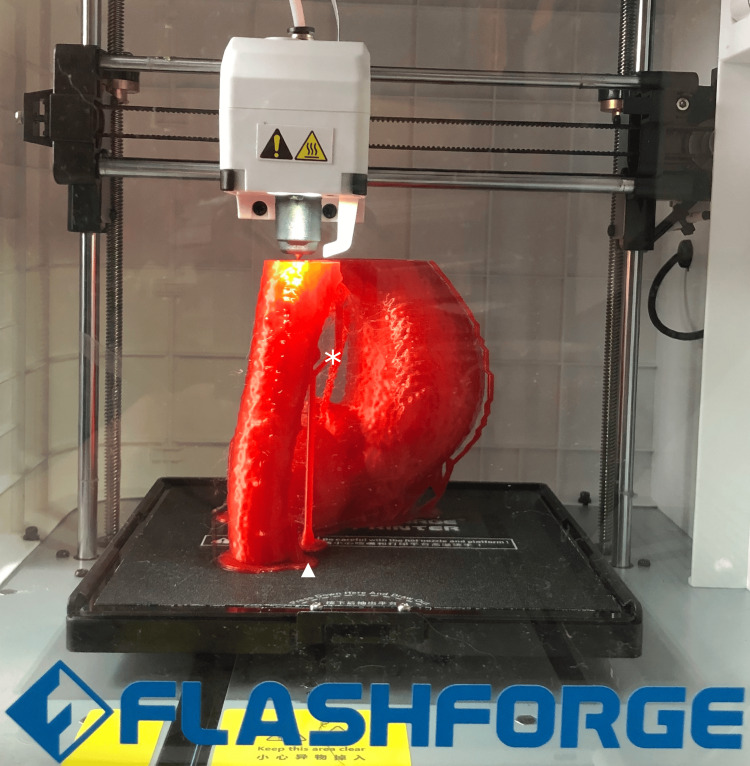
A model of an aneurysmal aorta being printed within the Flashforge Adventurer 3 FDM 3D printer. Note the presence of rafts on the printer base and supports. ★ Indicates *temporary support structures* used to maintain overhanging geometry during printing. ▲ Denotes the *raft*, a foundational base layer printed beneath the model to improve bed adhesion and reduce warping. The figure is the authors' own.

We print the model using polylactic acetate (PLA), which is cost-effective, environmentally friendly, and compostable, has a good surface finish, and is easy to print (Figure 3) [1]. Our standard print settings are a nozzle temperature of 210°C, bed temperature of 50°C, print speed of 60 mm/s, and layer height of 0.18 mm. Then, we print with rafts and supports enabled. With this layer height, each slice of the CT scan is printed in three to four layers, and the scaled Poisson surface redistribution aids with the transition between slices, especially where there would be a slight overhang. 

Following printing, the rafts and supports are simply broken off.

We have also used ABS on occasion, which is more sensitive to print settings, creates an odour while printing, and is less environmentally friendly. Typical print times range from six to 12 hours, depending on the size of the mesh. Aneurysmal aortas may take longer, due to the larger volume of material that is deposited to match the larger dimensions of the pathology.

For cases where a 3D-printed structure is used for intraoperative planning, we use a Formlabs Form 3B+ 3D Printer (Formlabs Inc., United States), which prints a biocompatible resin using an SLA technique. We use Formlabs Biomed Amber Resin, which is able to be sterilised by multiple techniques, including autoclaves [16]. This 3D printer creates items that may be safely sterilised and taken into the operative field and used for intraoperative planning, as described later. The SLA process is more time-consuming and requires additional steps. After printing, the item must be washed and cured prior to use. This process is also significantly more costly in terms of consumables.

## Discussion

Many surgeons and researchers have become more familiar with 3D printing and its applications in recent years, as the availability of the necessary hardware has improved, and prices have decreased. Research continues to advance into this space [17]. Consumer-level printers are within reach of many and have provided benefits to clinicians and educators alike.

3D printing in aortic surgery: enhancing visualisation and surgical planning 

The application of 3D printing in aortic surgery has revolutionised preoperative planning by providing tangible, patient-specific models. These models facilitate a deeper understanding of complex aortic anatomies, including aneurysms and dissections, which is crucial for determining graft extents and cross-clamp sites. Studies have demonstrated that 3D-printed aortic models can replicate intricate details such as intimal flaps and vessel tortuosity, aiding in surgical decision-making and potentially reducing operative times [18].

Moreover, the use of 3D-printed models has been shown to improve patient education and consent processes [3]. By providing a physical representation of the pathology, patients can better comprehend their condition and the proposed surgical intervention, leading to improved understanding and reduced preoperative anxiety.

**Figure 4:**
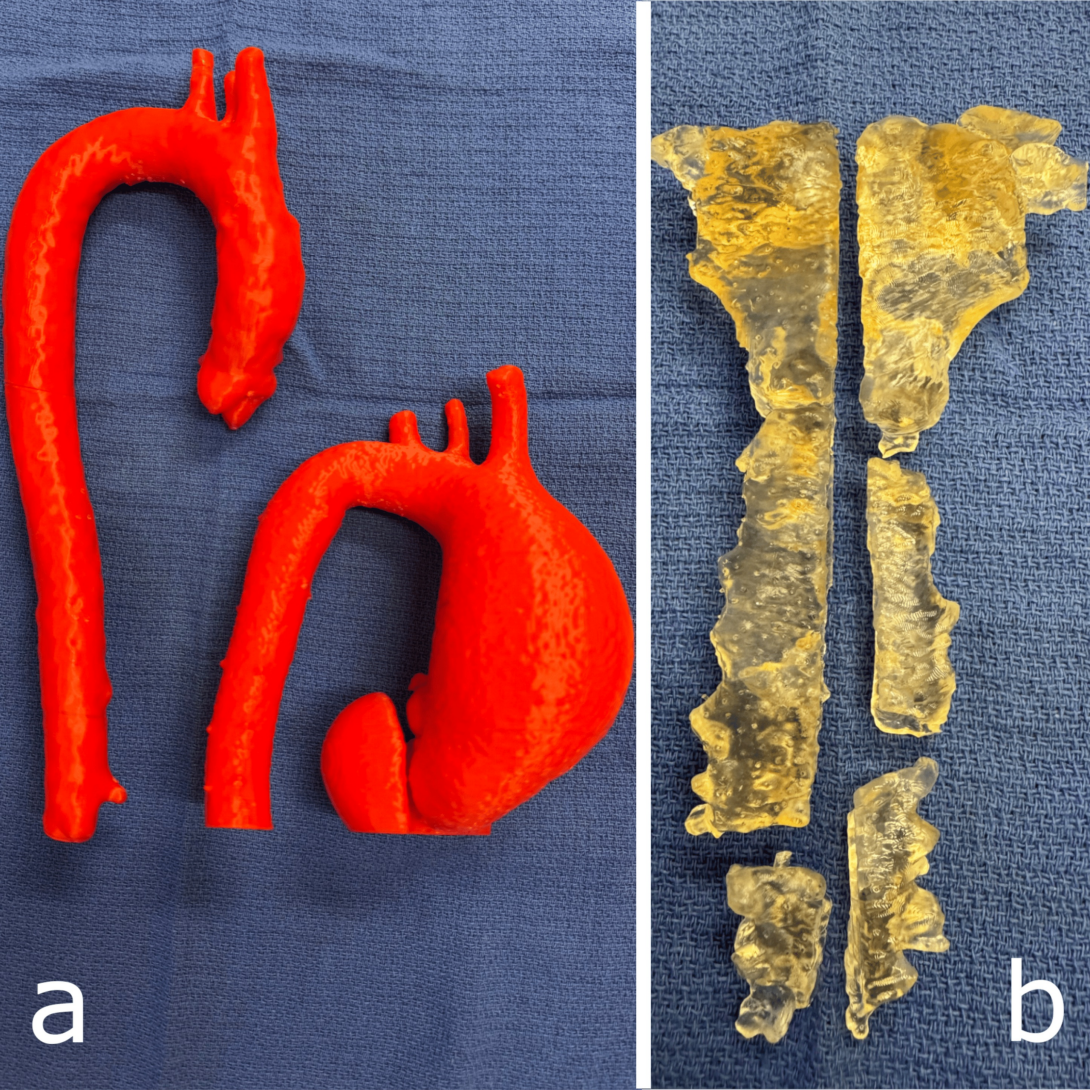
Examples of prints used in patient consent, education, and operative planning. Panel a) demonstrates two aortas, one with normal anatomy and dimensions and one with a large ascending aortic aneurysm. Panel b) shows the bony structure of the sternum post osteomyelitis and sternal dehiscence, printed in a biocompatible resin that is able to be sterilised and brought into the operative field for planning and plate pre-bending. The figure is the authors' own.

Figure 4 demonstrates examples of prints that we have used for consent, education, and operative planning. We have examples of normal aortic anatomy and aneurysmal aortas, which greatly help patients and students understand the pathology. Models of aneurysmal aortas also aid in preoperative planning of the extent of replacement, cannulation sites, and safe cross-clamp location. An example of our 3D printed models is shown in Figure 4.

Sternal and rib reconstruction: planning and advancements with biocompatible 3D printing

Sternal wound complications, such as dehiscence and osteomyelitis, pose significant challenges in cardiothoracic surgery. The integration of 3D printing has enabled the creation of customised, biocompatible sternal implants that conform precisely to patient anatomy. These implants, often fabricated from materials like titanium or porous polyethene, provide structural integrity and promote tissue integration [19].

In our practice, we use biocompatible resins to produce sterilisable sternal models, which can be brought into the sterile field to pre-bend sternal plates. We have used this technique with products such as those from the Synthes titanium sternal plate system (DePuy Synthes, Titanium Sternal Fixation System, Synthes GmbH, Switzerland) to reconstruct sternums in the unfortunate event of sternal dehiscence following cardiac surgery. An example of our model is shown in Figure 4, panel b). This approach enhances the accuracy of hardware placement and may reduce intraoperative time and complexity. 

Educational applications: addressing the decline in anatomical specimens 

**Figure 5:**
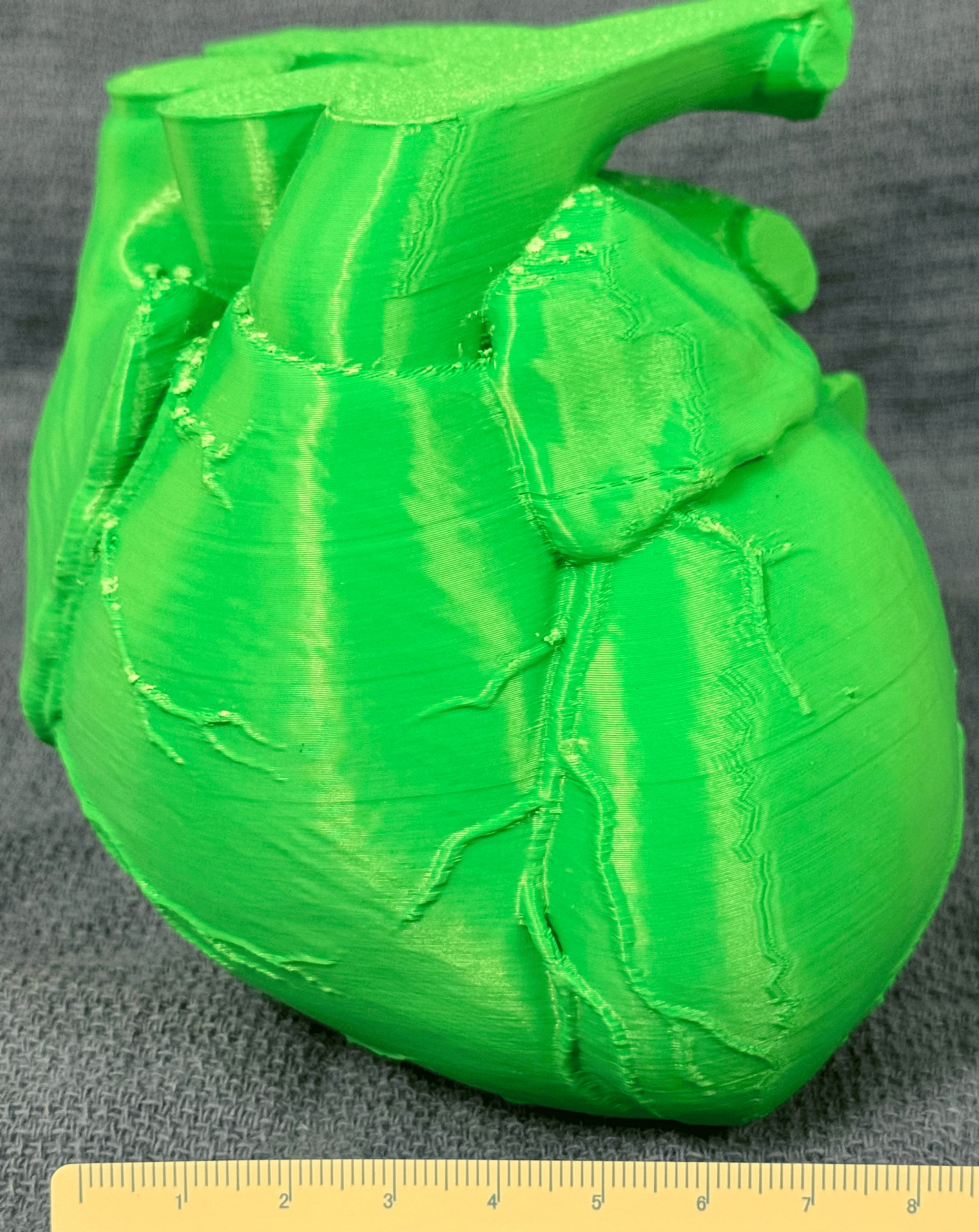
A full-sized model of a human heart has been useful for improving patient and student understanding of the size, surface anatomy, and features of this organ. The figure is the authors' own.

The scarcity of cadaveric specimens has impeded anatomical education in recent years [4]. 3D printing offers a viable alternative by producing accurate, durable models of human anatomy. Specifically, printing the first ribs allows for detailed study and comparison with other ribs, facilitating a better understanding of thoracic anatomy among medical students and residents. True-sized models of the human heart can be printed to aid in the understanding of surface anatomy and important structures, as shown in Figure 5. These models can be selected and customised to highlight specific anatomical features or pathologies, enhancing our ability to teach relevant surgical anatomy using substitutes for human specimens, especially where access to a university anatomy laboratory is difficult or impossible in a clinical setting. 

Workflow integration: from imaging to physical models 

Our workflow begins with high-resolution imaging, typically CT scans, which are converted into 3D digital models using specialised software. Our software toolchain consists of FOSS applications, which improve ease of access for clinicians [20]. These models are then printed using materials selected based on the intended application-rigid plastics for structural models or resins for simulating bone for operative planning and plate pre-bending. The entire process, from image acquisition to final print, can be completed within a timeframe that aligns with preoperative planning schedules, ensuring the timely availability of models for surgical preparation.

## Conclusions

Using this workflow, we are able to print thoracic anatomical structures with both FDM and SLA 3D printing techniques. This has aided us immensely in patient understanding and consent, operative planning, and teaching of medical students and residents. The models we have generated are high fidelity, anatomically accurate, and clinically relevant. Especially in the case of our FDM printer, the hardware required is consumer-grade, affordable, and uses environmentally friendly, cost-efficient consumables. Using our toolchain (largely consisting of FOSS applications), our methods are achievable by the majority of clinicians and could form a valuable adjunct to their own teaching, operative planning, and consent processes.
